# Co-Exposure of Microplastics and Avermectin at Environmental-Related Concentrations Caused Severe Heart Damage Through ROS-Mediated MAPK Signaling in Larval and Adult Zebrafish

**DOI:** 10.3390/toxics14010024

**Published:** 2025-12-25

**Authors:** Guanghua Xiong, Min Lu, Yaxuan Jiang, Huangqi Shi, Jinghong Liu, Xinjun Liao, Huiqiang Lu, Yong Liu, Gaoxiao Xu

**Affiliations:** 1 Key Laboratory of Embryo Development and Reproductive Regulation of Anhui Province, College of Biology and Food Engineering, Fuyang Normal University, Fuyang 236041, China; xionggh@fynu.edu.cn (G.X.); luminjy213@163.com (M.L.); jiangyaxuan2024@163.com (Y.J.); m15551882515@163.com (H.S.); 18800465208@163.com (J.L.); yong_liu2023@126.com (Y.L.); 2 Clinical Medical Research Center of Jinggangshan University, College of Life Sciences, Jinggangshan University, Ji’an 343009, China; xinjun_liao2022@126.com (X.L.); huiqiang_lu@yeah.net (H.L.)

**Keywords:** polystyrene microplastics (PS-MPs), avermectin (AVM), heart damage, oxidative stress, MAPK signaling, zebrafish

## Abstract

The widespread presence of polystyrene microplastics (PS-MPs) and agricultural pollutants such as avermectin (AVM) in aquatic environments poses a significant threat to aquatic organisms. However, the combined toxic effect of PS-MPs and AVM on cardiac development remains poorly understood. This study aimed to investigate the cardiac toxicity of AVM co-exposed with two sizes of MPs (large MPs, LMPs, 20 µm; small MPs, SMPs, 80 nm) in both larval and adult zebrafish. Firstly, under the co-exposure conditions of MPs and AVM, we observed significant cardiac developmental toxicity, including decreased survival rate, body length, and hatching rate, as well as a significant reduction in the number of myocardial cells. Secondly, the number of neutrophils and antioxidant enzyme activities such as CAT and SOD were greatly decreased, while inflammatory cytokines such as TNF-α and IL8 were significantly increased after co-exposure in larval zebrafish. Thirdly, there was severe disorganization of cardiomyocytes and interstitial edema in adult zebrafish hearts under the co-exposure by histopathological examination. Our results suggest that cardiomyocyte proliferation was suppressed, but heart apoptosis level and anti-apoptotic genes were significantly increased in the AVM+MPs co-exposure. Additionally, transcriptome sequencing and bioinformatics analysis revealed that significant changes in differentially expressed genes in the AVM+SMPs co-exposure group, particularly in the processes related to oxidation–reduction, inflammatory response, and the MAPK signaling pathway in the adult zebrafish heart. Furthermore, our pharmacological experiments demonstrated that inhibiting ROS and blocking the MAPK signaling pathway could partially rescue the heart injury induced by AVM and MPs co-exposure in both larval and adult zebrafish. In summary, this study suggested that co-exposure to AVM and MPs could induce heart toxicity mainly via the ROS-mediated MAPK signaling pathway in zebrafish. The information provided important insights into the potential environmental risk of microplastic and pesticide co-exposure on aquatic ecosystems.

## 1. Introduction

Polystyrene microplastics (PS-MPs) are common and novel environmental pollutants that have received widespread attention worldwide in recent years [1,2]. PS-MPs have the physicochemical characteristics of small size (usually <5 mm), large specific surface area, strong persistence, low degradation, and high hydrophobicity, which can be released into the environment and accumulate in the aquatic organisms for a long time [3,4]. Environmental monitoring studies have reported PS-MPs concentrations ranging from ng/L to µg/L in freshwater systems, with higher levels in contaminated areas [5]. Due to their frequent occurrence in water bodies, soil, and the atmosphere, the potential harm of PS-MPs to ecosystems and human health has increasingly become a current research hotspot [4,6]. Previous studies have suggested that PS-MPs can be transmitted through the food chain and bioaccumulate in aquatic and mammalian species, leading to various toxic effects, including immune inflammation, oxidative stress, and cell apoptosis [7]. Although numerous studies have explored the ecological toxicity and biological effects of PS-MPs, their impact on specific organ systems (such as the heart) and their combined toxicity mechanisms with other absorbed pollutants still require further investigation.

Long-term pesticide exposure can impair the human body and disturb the function of many important organs, such as the heart [8]. Avermectin (AVM) is a broad-spectrum anti-parasitic reagent widely used for pest control in agriculture and animal husbandry [9]. However, the environmental residues of AVM and their potential negative effects on non-targeted aquatic organisms have attracted widespread attention [10,11]. Typical environmental concentrations of AVM in surface waters range from ng/L to low µg/L, though higher levels may occur near agricultural runoff sites [12]. For example, AVM at environmentally realistic concentrations can enter into ecosystems through water bodies and induce significant ROS generation, DNA damage, and apoptosis in aquatic organisms [13,14]. Meanwhile, AVM exposure has been shown to cause various adverse effects such as neurotoxicity, reproductive toxicity, and immunotoxicity in zebrafish models [15,16]. Moreover, AVM is readily adsorbed by organic particles like microplastics and nanoplastics, which may further aggravate the harmful effects on the aquatic environment [17]. Therefore, exploring the combined toxic effects of AVM with other environmental pollutants such as PS-MPs on the heart organ is of great significance.

In recent years, zebrafish models have been shown to have significant advantages and have been widely used to study the aquatic toxicology of environmental pollutants on cardiac development [18,19]. Previous studies have demonstrated that PS-MPs can induce cardiac malformations, reduce heart rate, and alter expression of cardiac development genes in zebrafish [20]. Similarly, AVM has been shown to cause cardiotoxicity in aquatic organisms, including altered heart morphology and function [21]. Zebrafish is an ideal model organism characterized by transparent embryos, rapid development, a heart structure highly similar to mammals, and ease of gene editing and phenotype analysis [22]. In addition, zebrafish have been used to study the combined toxic effects of MPs and other pollutants such as heavy metals and pesticides. For example, co-exposure of MPs and sulfamethoxazole (SMZ) induced liver damage and promoted hepatocyte apoptosis through the ROS-mediated MAPK signaling pathway in zebrafish [23]. Co-exposure to MPs and arsenic damaged the reproductive system and adversely affected offspring growth and development in zebrafish [24]. Most studies indicated that co-exposure of MPs with other pollutants may increase their bioavailability through adsorption, thereby exacerbating their toxic effects [25,26]. However, the cardiotoxicity of MPs and AVM in zebrafish is still limited and requires further exploration.

The aim of this study is to investigate the effects and molecular mechanisms of abnormal cardiac development under co-exposure of PS-MPs and AVM in zebrafish. By combining environmental toxicology and molecular biology, we systematically evaluated the cardiotoxic effects of PS-MPs and AVM exposure alone and in combination on zebrafish heart development, oxidative stress, immune responses, and associated regulatory pathways. In summary, we have systematically studied the synergistic effect of PS-MPs and AVM co-exposure on zebrafish cardiotoxicity, as well as combined high-throughput RNA-Seq and pharmacological rescue experiments to reveal the molecular mechanism of its cardiotoxicity. This study provides a new theoretical basis for evaluating the potential environmental risks of co-exposure of nanomaterials and adsorbed pollutants on the heart in aquatic organisms.

## 2. Materials and Methods

### 2.1. Chemical Reagents and Characterization of PS-MPs

Avermectin was purchased from Sigma-Aldrich (>95% purity, Cas no. 71751-41-2, Saint Louis, MO, USA), which was initially dissolved in dimethyl sulfoxide (DMSO) and obtained a stock solution concentration of 100 mg/L. The PS-MPs of 80 nm (Cas no. 6-1-0005), 20 µm (Cas no. 6-1-2000), and green fluorescent microspheres (Cas no. 7-3-0005) were purchased from Baseline ChromTech (Tianjin, China). Although environmental MPs often exhibit irregular shapes, we used spherical PS-MPs to ensure uniformity in size and surface properties, which allows for reproducible exposure conditions and clearer interpretation of size-dependent effects. The MAPK inhibitor of Ralimetinib dimesylate (Cas no. HY-13241) and the ROS inhibitor of Histamine dihydrochloride (Cas no. HY-B0722) were purchased from MedChemExpress Biotech Co., Ltd. (MCE, Plainfield, NJ, USA). All other chemical reagents used in this study were of analytical grade.

The physical characterization of PS-MPs was analyzed by Zhongke Baice Information Technology Co., Ltd. (Beijing, China). Briefly, the morphological features and dispersion properties of two distinct sizes of microplastics, namely small microplastics (SMPs) and large microplastics (LMPs), were characterized using scanning electron microscopy (SEM; Hitachi SU8020, Tokyo, Japan). The surface charge characteristics of these microplastics were evaluated through zeta potential measurements performed on a Zetasizer Nano ZS90 instrument (Malvern Panalytical, Malvern, UK). The chemical composition and functional groups of the microplastic samples were analyzed by FT-IR using a Nicolet IS10 spectrometer (Thermo Scientific, Waltham, MA, USA).

### 2.2. Zebrafish Breeding and Embryo Collection

We used wild-type AB and transgenic zebrafish *Tg (myl7: GFP)* and *Tg (Lyz: dsRed)*. The zebrafish were maintained at 28 ± 1 °C with a 14:10 h light–dark cycle in a recirculating aquaculture system. Water quality parameters (temperature: 28 ± 1 °C; pH: 7.2 ± 0.2; dissolved oxygen: >6 mg/L; conductivity: 500 ± 50 μS/cm; ammonia: <0.05 mg/L; nitrite: <0.1 mg/L; nitrate: <5 mg/L) were monitored daily throughout the exposure period. Adult specimens were fed fairy shrimp (Artemia sp., INVE Aquaculture, Ghent, Belgium), while larvae received specialized feed (Gemma Micro 75, Skretting, Stavanger, Norway) twice daily. For embryo collection, sexually mature fish were separated in spawning boxes overnight, followed by baffle removal to initiate spawning. Fertilized eggs were subsequently transferred to 10 cm dishes and incubated at 28 °C under controlled light conditions. All experimental protocols were approved by the Animal Ethics Committee of Jinggangshan University (JGSU-202301003) and conducted in compliance with ethical guidelines.

### 2.3. Co-Exposure Assays of AVM and PS-MPs in Larval Zebrafish

The combined heart toxicity of AVM and small-sized microplastics (SMPs) as well as large-sized microplastics (LMPs) in larval zebrafish was investigated. For larval zebrafish treatment, *Tg (my17: GFP)* zebrafish embryos at 6 hpf were exposed to the following conditions: the control group, 50 µg/L of AVM, 50 µg/L of AVM combined with 100 µg/L of 20 µm microplastics (LMPs), and 50 µg/L of AVM combined with 100 µg/L of 80 nm microplastics (SMPs). The selected concentrations of AVM and MPs were higher than typical environmental levels to ensure detectable toxicological responses within the experimental timeframe. This approach is commonly used in mechanistic toxicology studies to elucidate pathways of toxicity that may also be relevant at lower, environmentally realistic concentrations. Each group contains at least three biological replicates, and each replicate contains at least 40 zebrafish. The dead zebrafish embryo was removed, and the survival rate was calculated for one week. Meanwhile, the development-related parameters such as body length and hatching rate at 72 hpf were also statistically counted in each group.

To observe the enrichment level of microplastics in zebrafish heart tissue, AB strain zebrafish (5 days post-fertilization, dpf) were exposed to green fluorescent PS-MPs for 72 h. After exposure, zebrafish were dissected to obtain heart organs, and the distribution and enrichment of green fluorescent microplastics in the heart tissue were observed using the inverted fluorescence microscope (Leica M165 FC, Wetzlar, Germany). In addition, in order to further investigate the toxic effects of co-exposure of AVM and PS-MPs on zebrafish hearts, transgenic zebrafish *Tg (my17: GFP)* were divided into the above four groups and continuously exposed for 72 h. After exposure, the heart rates were manually counted, and the zebrafish larvae were fixed with 1% low melting point agarose and observed for changes in cardiac phenotype under a fluorescence microscope (Leica M205FA, Wetzlar, Germany). Subsequently, the zebrafish were fixed with 4% PFA, and the number of myocardial cells was counted using Leica LAS X (V 4.7.0) software. The average number of myocardial cells from at least 20 zebrafish was calculated in each group, which specifically expressed green fluorescent protein (GFP).

### 2.4. Intraperitoneal Injection of AVM and PS-MPs in Adult Zebrafish

To examine the cardiotoxicity of AVM and PS-MPs co-exposure in adult zebrafish, we performed intraperitoneal injections. We microinjected 10 µL of each of the aforementioned substances into the abdominal cavity of 6-month-old adult zebrafish daily for one week. Prior to injection, zebrafish were anesthetized with 0.02% tricaine methanesulfonate (MS-222, Sigma-Aldrich). At the end of the experiment, the adult zebrafish were euthanized with an overdose of MS-222, and heart tissue from each group was harvested, weighed (average tissue weight: 5–7 mg), and preserved at −80 °C for subsequent analysis.

### 2.5. Quantitation of Neutrophils in Larval Zebrafish After MPs and AVM Co-Exposure

To investigate neutrophil dynamics after AVM and MPs co-exposure, we utilized *Tg (Lyz: dsRed)* zebrafish lines that enable precise labeling of these immune cells. At 72 hpf, the zebrafish embryos were exposed to different experimental conditions, and the spatial organization of fluorescently labeled neutrophils in the caudal hematopoietic tissue (CHT) was then visualized using a fluorescence stereomicroscope (Leica M205FA, Wetzlar, Germany). For quantitative assessment of neutrophils in each group, we employed ImageJ 6.0 software (NIH, Bethesda, MD, USA) to process the captured images that focused on specific regions of interest. The analytical approach included three key parameters: measurement of fluorescence intensity beyond a defined threshold, particle counting, and quantification of the immune cells.

### 2.6. Total RNA Extraction and Real-Time PCR

Total RNA was isolated from heart tissues using TRIzol reagent (Invitrogen, Waltham, MA, USA). Following chloroform separation and isopropanol precipitation, RNA was washed with 75% ethanol and quantified using NanoDrop 2000 (Waltham, MA, USA). cDNA was synthesized from 1 μg RNA with the PrimeScript RT Kit (Takara, Osaka, Japan). qPCR was performed using SYBR Premix Ex Taq II (Takara) on a QuantStudio 6 Flex system. Cycling conditions were 95 °C for 30 s, then 40 cycles of 95 °C for 10 s and 60 °C for 30 s. Specificity was confirmed by melt curve analysis. Gene expression was calculated using the 2^−ΔΔCt^ method with β-actin as the endogenous control. All reactions were run in triplicate. Primer sequences are provided in Table S2.

### 2.7. Detection of the Antioxidant Activities

Cardiac tissues obtained from zebrafish (approximately 5–7 mg) were subjected to homogenization in an ice-cold phosphate buffer (pH 7.4) using a tissue homogenizer (TissueLyser LT, Qiagen, Germany). Subsequently, the homogenates underwent centrifugation at 12,000× *g* for 15 min at 4 °C using a Centrifuge 5430R (Eppendorf, Hamburg, Germany), after which the supernatants were collected for further enzymatic assays. The activities of antioxidant enzymes, including catalase (CAT) and superoxide dismutase (SOD), as well as lactate dehydrogenase (LDH), were measured employing commercial assay kits from the Nanjing Jiancheng Bioengineering Institute in China, adhering to the protocols specified by the manufacturer. Protein concentrations were determined via the BCA method [27], using bovine serum albumin as a standard reference.

### 2.8. Histopathological Analysis

Heart tissues were fixed in 4% PFA, processed through graded ethanol, paraffin-embedded, and sectioned at 5 μm. Sections were stained with (1) H&E, (2) Masson’s trichrome to visualize collagen, and (3) PAS for glycogen/mucopolysaccharides, following established protocols. Images were acquired using a Nikon Eclipse E100 microscope and quantitatively analyzed with ImageJ 6.0.

### 2.9. Cell Apoptosis and Cell Proliferation Analysis

For proliferation analysis, OCT-embedded heart sections were stained with an anti-PCNA antibody (1:1000, Sigma, Saint Louis, MO, USA) and counterstained with DAPI, following a previous method [28]. Images were captured using a Leica TCS SP8 confocal microscope. Apoptosis was assessed by TUNEL staining (Transgene, Cat# FA201, Beijing, China) according to the manufacturer’s instructions, with nuclei counterstained by DAPI. TUNEL-positive cells were quantified using ImageJ.

### 2.10. Transcriptome Sequencing and Bioinformatics Analysis

Total RNA was extracted with TRIzol, and quality was verified (RIN > 8.0). mRNA libraries were prepared using the NEBNext Ultra RNA Library Prep Kit and sequenced on an Illumina NovaSeq 6000 (150 bp paired-end). After quality control (FastQC, Trimmomatic), reads were aligned to the GRCz11 genome with HISAT2. Gene expression was quantified using StringTie, and differentially expressed genes (DEGs) were identified with DESeq2 (|log_2_FC| > 1, adjusted *p* < 0.05). DEGs were hierarchically clustered and compared across groups using Venn analysis. Functional enrichment of KEGG pathways and GO terms was performed with DAVID (*p* < 0.05). DEGs related to MAPK signaling and oxidation–reduction were further classified via KEGG. Results were validated by BLAST (V. 2.15.0) against the NCBI NR database.

### 2.11. Molecular Docking Analysis

Docking between AVM and MAPK1 was performed using AutoDock Vina (V. 1.2.5). The 3D structure of AVM was obtained from PubChem (CID: 6858006), and that of MAPK1 (NP_878308) was retrieved from NCBI. The binding site was defined within a 15 Å radius of the ligand. The conformation with the lowest binding energy (kcal/mol) was selected for analysis. Electrostatic potential (ESP) and LUMO were computed as toxicity-related descriptors. Final docking poses were visualized using PyMOL (V.3.0).

### 2.12. The Pharmacological Rescue Experiments

Pharmacological rescue experiments were performed using the ROS inhibitor N-acetylcysteine (NAC) and the MAPK inhibitor SB203580. After inducing cardiac injury with a 72 h co-exposure to AVM and SMPs, both larval and adult zebrafish were treated with NAC or SB203580 for an additional 72 h. Rescue effects were assessed by (1) qPCR analysis of MAPK pathway genes (*JNK1, ERK1*) and cardiac/inflammatory markers; (2) measurement of ROS levels (DCFH-DA assay [29]) and SOD activity; (3) fluorescence imaging of heart morphology in *Tg (myl7: GFP)* larvae; and (4) TUNEL staining to quantify cardiomyocyte apoptosis in adult heart tissues.

### 2.13. Statistical Analysis

Statistical analyses were conducted using GraphPad Prism (V.10.2.1), with results presented as mean ± SD. Data normality and variance homogeneity were confirmed using Shapiro–Wilk and Levene’s tests, respectively. Group comparisons were performed by one-way ANOVA (*p* < 0.05), followed by Tukey’s post hoc test with Bonferroni correction for multiple comparisons.

## 3. Results

### 3.1. The Physical Characterizations of PS-MPs

**Figure 1:**
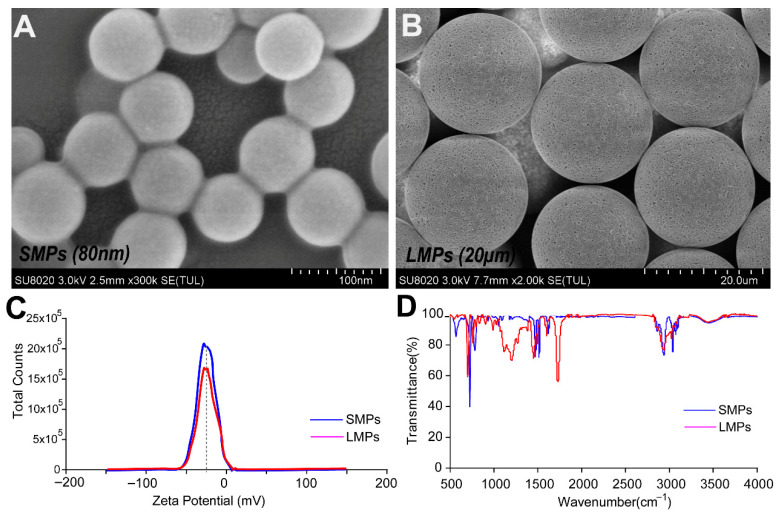
Physical characterization of two different sizes of PS-MPs. (**A**) The surface morphology characteristics of 80 nm small-sized microplastics (SMPs) were detected using scanning electron microscopy (SEM). Scale bar = 100 nm. (**B**) The image of 20 µm large-sized microplastics (LMPs) was also acquired by SEM. Scale bar = 20 µm. (**C**) The zeta potential of SMP and LMP nanomaterials was measured by the Zetasizer instrument. (**D**) The functional chemical groups of SMPs and LMPs were detected by Fourier-transform infrared spectroscopy (FT-IR) analysis.

The physical properties of two PS-MP sizes (80 nm, SMPs; 20 μm, LMPs) were characterized. SEM revealed that SMPs were uniform nanospheres, while LMPs showed microporous spherical structures (Figure 1A,B). Zeta potential was approximately −45.2 mV in ultrapure water, with SMPs exhibiting higher particle counts than LMPs (Figure 1C). FT-IR confirmed polystyrene as the primary component, with spectral differences between sizes (Figure 1D). The results indicate that both PS-MPs were well-dispersed and stable, suitable for zebrafish exposure studies.

### 3.2. Co-Exposure of MPs and AVM Aggravated the Heart Damage in Larval Zebrafish

**Figure 2:**
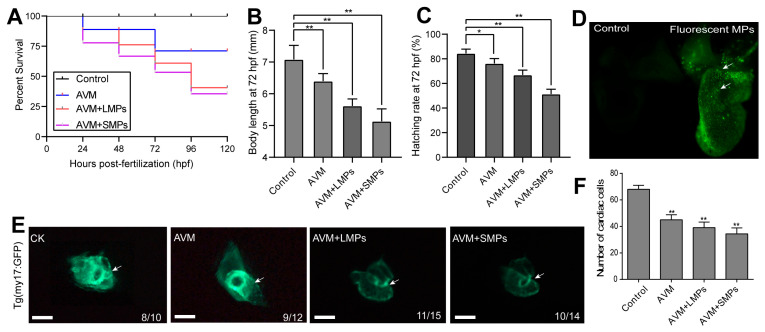
The co-exposure of AVM and PS-MPs caused developmental disorders and heart injury in zebrafish embryos. (**A**) The survival rate of zebrafish embryos in response to AVM and MPs treatment. (**B**) The body length of zebrafish larvae at 72 hpf after AVM and MPs treatment was calculated in each group (n = 30). (**C**) The hatching rate of zebrafish larvae at 72 hpf was analyzed in each group (n = 30). (**D**) The enrichment characteristics of fluorescent MPs in zebrafish heart tissues. The white arrow indicates the area where GFP-MPs appeared. (**E**) The cardiac developmental toxicity was observed in *Tg (my17: GFP)* zebrafish embryos after AVM and MPs co-exposure (n = 20). Scale bar = 200 µm. (**F**) The number of cardiac cells was quantified in each group using the ImageJ software. Data are presented as the mean ± SD (n = 4), Student’s *t* test, * *p* < 0.05, ** *p* < 0.01.

As shown in Figure 2A, the survival rates of zebrafish exposed to AVM (50 µg/L), MPs (100 μg/L), AVM + large-sized microplastics (AVM+LMPs, 20 µm), and AVM + small-sized microplastics (AVM+SMPs, 80 nm) for 72 h were 72.7 ± 3.1%, 65.6 ± 5.3%, and 54.4 ± 4.8%, respectively. Compared with the control group, the survival rate of the AVM+SMPs group was significantly reduced (*p* < 0.01), indicating that co-exposure of small-sized microplastics with AVM is more toxic to zebrafish embryos. The body length showed that co-exposure of AVM and PS-MPs had a more significant inhibitory effect on the growth and development of zebrafish embryos (Figure 2B). Similarly, the hatching rate of the AVM+SMPs group had the lowest value, indicating that the co-exposure of small-particle-size MPs and AVM had a more significant inhibitory effect on cardiac function (Figure 2C).

To further demonstrate the entry and distribution of microplastics into zebrafish, we exposed AB-type zebrafish to GFP fluorescent MPs. The results showed that green fluorescent microplastics were significantly enriched in zebrafish heart tissue observed by fluorescence microscopy (Figure 2D). In addition, our findings revealed a significant decrease in fluorescence intensity in the AVM group compared to the control group using *Tg (my17: GFP)* larval zebrafish. In the co-exposure groups, cardiac developmental abnormalities were more significant, manifested as a significant reduction in cardiac lumen and worsening of pericardial edema (Figure 2E). Further statistical analysis showed that the number of myocardial cells in the AVM, AVM+LMPs, and AVM+SMPs exposure groups were significantly lower than those of the control group (Figure 2F). Taken together, the synergistic effect of microplastics, especially small-particle MPs, with environmental pollutants may have more serious negative impacts on the heart development and function in zebrafish.

### 3.3. Co-Exposure Caused a Synergistic Toxic Effect on the Immune and Antioxidant Defense System

**Figure 3:**
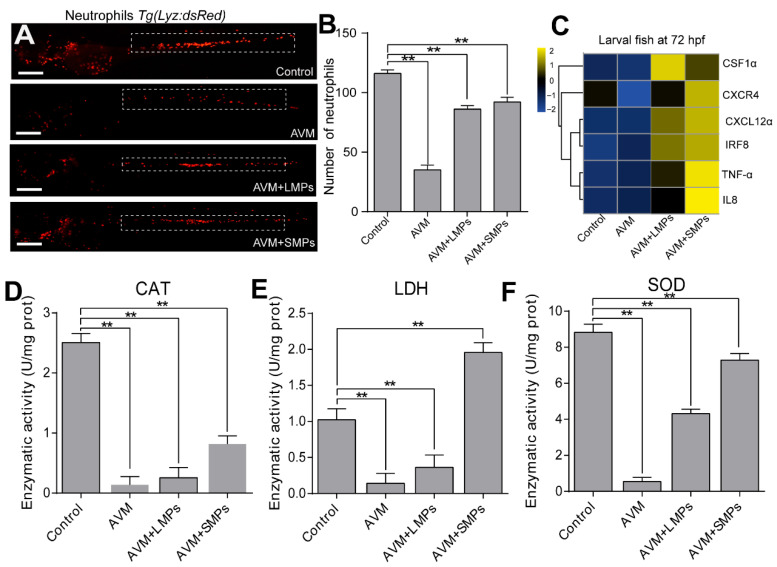
Co-exposure of AVM and MPs induced an immune inflammatory response and oxidative stress in zebrafish embryos. (**A**) The representative images of *Tg (Lyz: dsRed)* neutrophils in zebrafish at 72 hpf. Scale bar = 100 μm. (**B**) The number of neutrophils in each group was quantified using ImageJ software (n = 10). (**C**) The heatmap shows the mRNA levels of inflammatory cytokines under different treatments. (**D**) The antioxidant activities of CAT were detected in each group. (**E**) The antioxidant activities of LDH were detected in each group. (**F**) The antioxidant activities of SOD were detected in each group. Data are presented as the mean ± SD (n = 4), Student’s *t* test, ** *p* < 0.01.

The dynamic changes in innate immune cells such as neutrophils were observed using transgenic *Tg (Lyz: dsRed)* lines. The results showed that compared with the control group, the neutrophils in the caudal hematopoietic tissue (CHT) area of the zebrafish tail were significantly reduced under the AVM group, while the neutrophils in the two co-exposure groups (AVM+LMPs and AVM+SMPs) were slightly increased compared with the AVM group (Figure 3A). Quantitative analysis indicated that co-exposure of MPs and AVM significantly reduced the number of neutrophils in zebrafish embryos (Figure 3B). The results suggested that the relative mRNA expression of key immune inflammatory genes was significantly upregulated in the AVM+LMPs and AVM+SMPs exposure groups, with TNF-α, IL-8, and IRF8 expression increased in the AVM+SMPs group (Figure 3C).

To evaluate the changes in oxidative stress levels, we measured the antioxidant enzyme activity of zebrafish under different treatments. The results revealed that compared with the control group, the enzyme activities of CAT and SOD in all exposed groups were mostly reduced, especially in the AVM group, where the decrease was most significant (Figure 3D). Lactate dehydrogenase (LDH) activity was also measured as an indicator of cellular damage. In the AVM+LMPs and AVM+SMPs co-exposure groups, there was a partial increase in the enzyme activity of these enzymes compared to the AVM group; especially, LDH was higher in the AVM+SMPs group than in the control group. Altogether, these results indicated that the co-exposure of AVM and microplastics significantly altered the number of neutrophils, increased the expression of immune inflammatory genes, and inhibited antioxidant activities in zebrafish.

### 3.4. Intraperitoneal Injection of AVM and MPs Aggravated the Histopathology and Cardiomyocyte Apoptosis in Adult Zebrafish

**Figure 4:**
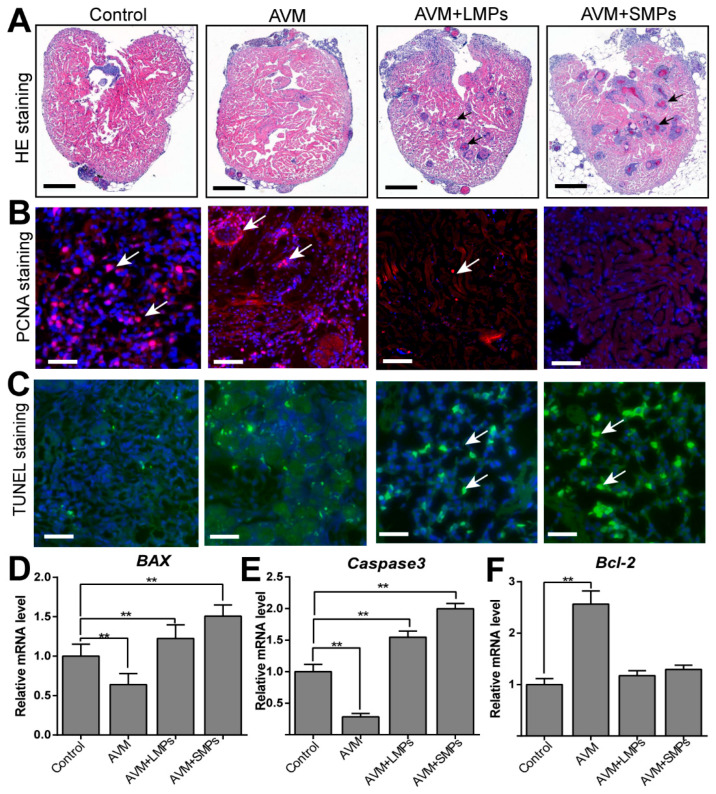
Co-exposure of AVM and MPs caused severe heart damage and myocardial apoptosis in adult zebrafish. (**A**) The pathological morphology of adult zebrafish heart tissue in each group was observed using H&E staining. Black arrows mark the lesion areas in the zebrafish heart. (**B**) The cardiomyocyte proliferation was evaluated in adult zebrafish heart tissues by PCNA immunofluorescence staining. Scale bar = 50 μm. (**C**) The cell apoptosis of adult zebrafish heart was analyzed in each group by using TUNEL staining. The white arrow indicates the apoptotic cardiomyocytes. (**D**–**F**) The relative mRNA levels of apoptosis genes such as BAX, Caspase 3, and Bcl-2 were measured by qRT-PCRs. Data are presented as mean ± SD (n = 4), Student’s *t* test, ** *p* < 0.01.

To assess the direct cardiotoxic effects of AVM and MPs in adult zebrafish, we performed intraperitoneal injections. The histopathological changes in zebrafish heart tissue were evaluated using H&E staining. In the control group, the heart tissue exhibited a normal structure with well-organized cardiomyocytes and intact myocardial fibers, while the AVM+LMPs and AVM+SMPs injection groups showed more severe damage, characterized by significant cardiomyocyte disarray, increased interstitial edema, and focal necrosis (Figure 4A). These results indicated that co-injection of AVM and MPs, especially small-sized MPs, significantly increased the risk of cardiac pathology in adult zebrafish. Moreover, the proliferation of cardiomyocytes in adult zebrafish hearts was evaluated using staining for proliferating cell nuclear antigen (PCNA). It is suggested that the control group exhibited a moderate number of PCNA-positive cells, while in the AVM+LMPs and AVM+SMPs groups, the number of PCNA-positive cells decreased significantly, with the AVM+SMPs group showing the lowest proliferative activity (Figure 4B).

Additionally, we also evaluated cardiomyocyte apoptosis after AVM and MPs injection using TUNEL staining. The findings indicated that the control group exhibited few TUNEL-positive cells, and the number of TUNEL-positive cells increased slightly in the AVM injection group. However, in the AVM+LMPs and AVM+SMPs groups, the number of TUNEL-positive cells increased significantly, with the AVM+SMPs group showing the highest apoptosis level (Figure 4C). Furthermore, the relative mRNA expression of pro-apoptotic genes (Bax and Caspase-3) increased by 1.8-fold and 2.3-fold, respectively (Figure 4D,E), while the anti-apoptotic gene Bcl-2 was significantly decreased in the AVM+SMPs group, indicating that small-sized MPs synergistically enhanced AVM-induced apoptosis and suppressed cardiomyocyte proliferation at the molecular level (Figure 4F). In summary, injection of AVM and MPs, particularly small-sized MPs, induced severe histopathological changes, myocardial fibrosis, and increased disease probability in zebrafish heart tissue.

### 3.5. RNA-Seq Analysis of DEGs and Functional Enrichment

**Figure 5:**
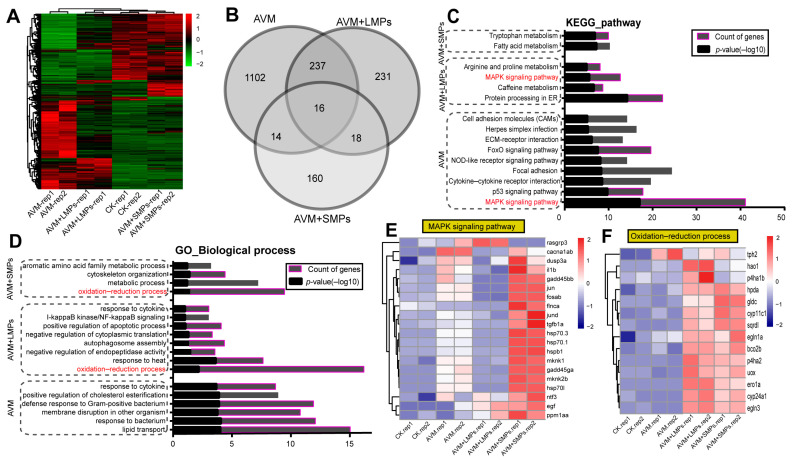
RNA-Seq identified the differentially expressed genes in zebrafish hearts after AVM and MPs co-exposure. (**A**) The hierarchical clustering analysis of DEGs in each group is presented by heatmap. Red represents significantly upregulated genes, while green represents significantly downregulated genes. (**B**) Venn diagram of differential genes in three groups. The number of overlapped genes appears in the corresponding region. (**C**) The KEGG pathway classification of all DEGs in the AVM, AVM+LMPs, and AVM+SMPs groups when compared with the control group. The X-axis presents the −log_10_ (*p*-value) or count of genes, and the top pathways are displayed in each group. (**D**) Functional enrichment analysis of all DEGs genes in GO-biological processes (GO-BPs). (**E**) The cluster analysis revealed distinct expression profiles of MAPK signaling pathway-associated genes in different groups. (**F**) The heatmap visualization demonstrates group-specific expression patterns of DEGs involved in oxidation–reduction signaling pathways.

Transcriptome analysis of adult zebrafish hearts after AVM and MPs exposure identified distinct sets of differentially expressed genes (DEGs): 1369 in AVM, 502 in AVM+LMPs, and 208 in AVM+SMPs groups (Supplementary Table S1). Hierarchical clustering revealed clear separation between control and treated samples (Figure 5A). Venn analysis showed 16 DEGs common to all groups (Figure 5B).

KEGG pathway enrichment highlighted the MAPK signaling pathway as the most significantly altered (Figure 5C). GO analysis indicated that DEGs were predominantly associated with the “oxidation–reduction process” in the co-exposure groups (Figure 5D). Key MAPK-related genes (*jund*, *tgfb1a*, *hsp70.3*) were upregulated 3.3- to 4.1-fold in the AVM+SMPs group (Figure 5E). Redox-related genes (*cyc11c1*, *gldc*, *egln1a*) were also elevated in co-exposure groups, suggesting interplay between MAPK signaling and oxidative stress (Figure 5F). Together, these results demonstrate that combined AVM and MPs exposure robustly activates immune and oxidative stress responses in the zebrafish heart.

### 3.6. ROS-Mediated MAPK Signaling Pathway Was Involved in Heart Damage in Zebrafish

**Figure 6:**
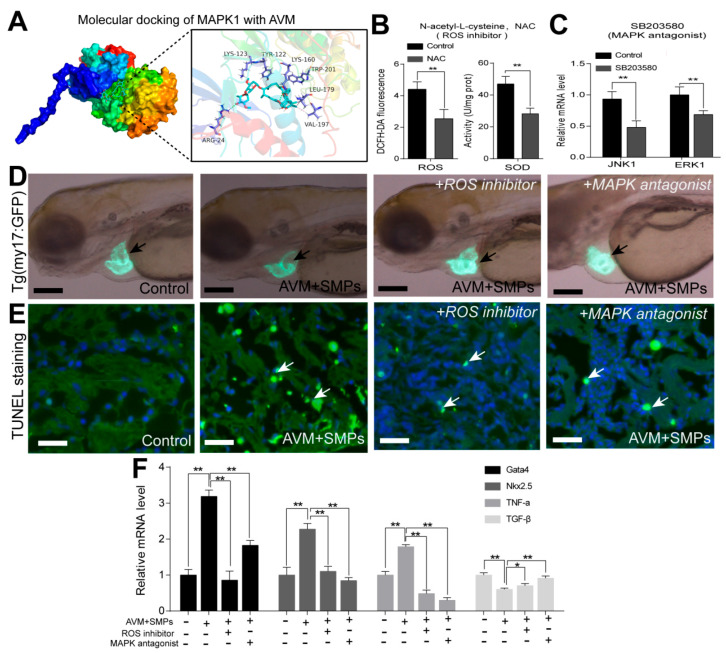
Inhibiting ROS and MAPK signaling could partially rescue the heart damage in both larval and adult zebrafish after AVM and MPs co-exposure. (**A**) The docking pose of AVM binding to MAPK1 protein was performed with the AutoDock Vina and visualized with the PyMOL software. (**B**) The antioxidant enzyme activities of ROS and SOD were detected in adult zebrafish hearts after ROS inhibitor (NAC) treatment. (**C**) The target gene expression was detected in adult zebrafish hearts after the MAPK signaling antagonist (SB203580) treatment. (**D**) The heart morphology of *Tg (my17: GFP)* larval zebrafish that were exposed to ROS inhibitor or MAPK antagonist conditions under the AVM+SMPs co-exposure. (**E**) The myocardial cell apoptosis was detected in adult zebrafish by TUNEL staining under various treated conditions. (**F**) The relative mRNA expression levels of key genes for cardiac development and immune inflammation were measured in each group. Data are presented as the mean ± SD (n = 4), Student’s *t* test, * *p* < 0.05, ** *p* < 0.01 compared with the AVM+SMPs group.

Molecular docking revealed a stable binding conformation between AVM and MAPK1, with a binding energy of −5.5 kcal/mol and interactions involving residues such as ARG-24, LYS-123, TYR-122, LYS-160, TRP-201, LEU-179, and VAL-197 (Figure 6A), indicating a direct interaction with the MAPK pathway. Pharmacological inhibition experiments confirmed the functional role of ROS and MAPK signaling in AVM+MPs-induced cardiotoxicity. Inhibition of ROS with NAC reduced ROS levels and SOD activity (Figure 6B), while MAPK inhibition with SB203580 downregulated *JNK1* and *ERK1* expression (Figure 6C). In larvae, co-treatment with NAC or SB203580 partially rescued the cardiac malformations induced by AVM+SMPs (Figure 6D). TUNEL staining showed that both inhibitors attenuated cardiomyocyte apoptosis in adult hearts (Figure 6E). Furthermore, the upregulation of cardiac development genes (*Gata4*, *Nkx2.5*) and the pro-inflammatory cytokine *TNF-α* under AVM+SMPs exposure was suppressed by NAC or SB203580, whereas the anti-inflammatory gene *TGF-β* was increased (Figure 6F). Together, these results demonstrate that AVM+MPs-induced heart injury is mediated through ROS-dependent activation of the MAPK signaling pathway in both larval and adult zebrafish.

## 4. Discussion

Microplastics are defined as plastic particles smaller than 5 mm, which pose significant harm by contaminating ecosystems, affecting aquatic organisms, and potentially entering the human food chain [30,31]. Research on microplastics and other absorbed pollutants is crucial for understanding their environmental distribution and adverse effects, thereby guiding the development of effective prevention strategies to protect ecological and human health [32,33]. Among the various pollutants found in marine environments, AVM and PS-MPs are of particular concern due to their detrimental consequences on both aquatic life and human health [34]. Nevertheless, there is still a lack of comprehensive knowledge concerning the toxic effects resulting from the combined exposure of MPs with varying particle sizes in aquatic organisms.

The significant decrease in heart rate and cardiomyocyte number in zebrafish exposed to AVM and SMPs suggests that small-sized MPs may have a more pronounced effect due to their higher bioavailability and ability to penetrate tissues more effectively. This is consistent with previous research indicating that smaller MPs can accumulate in organs more readily than larger particles, leading to greater biological impacts [35,36]. The increased oxidative stress, as evidenced by reduced antioxidant enzyme activities (CAT, SOD), further supports the hypothesis that MPs and AVM co-exposure disrupts cellular redox balance, leading to tissue damage. This finding is in line with studies showing that MPs can induce oxidative stress by generating reactive oxygen species (ROS), which in turn activates inflammatory pathways and apoptosis [37,38]. The upregulation of pro-inflammatory cytokines (TNF-α, IL-8) and the downregulation of anti-apoptotic genes (Bcl-2) in the AVM+SMPs group further corroborate the role of inflammation and apoptosis in the observed zebrafish cardiac damage.

The transcriptomic analysis revealed significant changes in gene expression related to the MAPK signaling pathway, oxidation–reduction, and immune response. The upregulation of genes such as jund, tgfb1a, and hsp70.3 in the AVM+SMPs group suggests that the MAPK pathway plays a central role in mediating the cardiac toxicity induced by AVM and MPs co-exposure. This is consistent with previous studies that have implicated the MAPK pathway in oxidative stress and inflammation-mediated tissue damage [39,40]. The molecular docking results further support this by showing a strong interaction between AVM and the MAPK1 protein, indicating that AVM may directly modulate MAPK signaling. The pharmacological rescue experiments further suggested the central role of ROS-mediated MAPK signaling in zebrafish heart injury. These findings are significant because they not only elucidate the molecular mechanisms underlying the combined toxicity of AVM and MPs but also suggest potential therapeutic targets for mitigating their effects.

In addition, our findings demonstrated that co-exposure to AVM and PS-MPs, particularly small-sized MPs (SMPs), significantly exacerbated cardiac toxicity in both the larval and adult zebrafish. The results align with previous studies that have highlighted the individual toxic effects of AVM and MPs on aquatic organisms, but this study provides novel insights into their combined effects, especially on cardiac development and function [41]. The observed reduction in heart rate, cardiomyocyte proliferation, and increased apoptosis in zebrafish exposed to AVM and MPs underscores the synergistic effects of these pollutants, which may have important implications for aquatic ecosystems and human health.

Taken together, the findings of this study have important implications for environmental risk assessment and the regulation of pollutants. The synergistic effects of AVM and MPs on cardiac development and function highlight the need for a more integrated approach to environmental monitoring and management. Current regulations often assess the toxicity of individual pollutants, but this study demonstrates that combined exposure to multiple pollutants can have more severe effects than exposure to a single pollutant. Therefore, regulatory frameworks should consider the combined effects of pollutants when setting safety thresholds and guidelines. Additionally, the identification of the ROS-mediated MAPK signaling pathway as a key mechanism of toxicity opens up new avenues for therapeutic interventions. Future research should include a broader range of species to better understand the ecological impacts of AVM and MPs co-exposure.

## 5. Conclusions

In conclusion, this study provides compelling evidence that co-exposure to AVM and MPs, particularly small-sized MPs, exacerbates cardiac toxicity in zebrafish. The findings underscore the importance of considering the combined effects of environmental pollutants and highlight the necessity for more comprehensive regulatory frameworks. Future research should focus on long-term exposure studies, transgenerational effects, and the development of therapeutic strategies to mitigate the toxic effects of these pollutants. By addressing these gaps, we can enhance our understanding of the ecological and health risks posed by AVM and MPs, thereby facilitating the development of more effective strategies for environmental protection and public health.

## Data Availability

The original contributions presented in this study are included in the article/Supplementary Materials. Further inquiries can be directed to the corresponding author.

## Supplementary Materials

The following supporting information can be downloaded at https://www.mdpi.com/article/10.3390/toxics14010024/s1, Table S1: The differentially expressed genes identified in various comparative conditions; Table S2: Sequences of the primer used in the qRT-PCRs.
